# A future in 3D: Analyzing morphology in all dimensions

**DOI:** 10.1093/plphys/kiac190

**Published:** 2022-04-27

**Authors:** Alexandra J Burgess, Mateusz Majda

**Affiliations:** Agriculture and Environmental Sciences, School of Biosciences, University of Nottingham, Loughborough, UK; Department of Computational and Systems Biology, John Innes Centre, Norwich, UK

Analysis of plant morphology is of interest across all scales of order; from molecule to cell through organism, field and even landscape levels (Burgess et al., 2017; Sapala et al., 2019; Strauss et al., 2021). Morphology is critical in determining function, and as such, accurately characterizing morphology in 3D is a major focus of many research programs. However, segmentation of 3D models is the most substantial challenge in analysis pipelines, and automated methods have been sought to improve the throughput of segmentation (Strauss et al., 2021).

For tissue-level morphology, shape is determined by cellular growth rates and proliferation along a designated axis (Whitewoods and Coen, 2017). Differences in cell division and cell expansion (i.e. asymmetric growth) lead to alterations in morphology due to changes in curvature (Rueda-Contreras et al., 2018; Zhou and Zhang, 2022). However, large-scale analysis of morphology in multicellular organisms requires an accurate estimate of the shape of all cells across several representative specimens. Capturing these structures is gaining traction through the generation of realistic 3D models through confocal laser scanning microscopy or light sheet fluorescence microscopy. However, the rapid and large-scale analysis of such models has been restricted due to the complexity of segmenting different tissue shapes (Wolny et al., 2020).

Plant tissues consist of a small number of different cell types arranged in distinct layers. Thus, analyzing the geometric and molecular parameters of such cells becomes feasible using automated approaches to differentiate the layers (Strauss et al., 2021). Up to now, this has been generally achieved through distance measurements and does not account for the growth axis (Montenegro-Johnson et al., 2019). However, as plant structures become more complex, such as through curvature, folds, or dome-shaped protrusions, automating cellular differentiation becomes more complex.

In this issue, Vijayan et al. (2022) present a tool for the automatic characterization and analysis of plant tissue morphology. By incorporating information on the organ axis and extracting the cells in different tissue layers based on the organ surface, the authors were able to automatically annotate cellular layers of even complex morphology; exemplified by ovule and funiculus (ovule stalk) development in Arabidopsis (*Arabidopsis thaliana*), the archegonium (female reproductive structure) of the model species the common liverwort (*Marchantia polymopha*), and the cup-shaped trap of the aquatic carnivorous plant the floating bladderwort (*Utricularia gibba*).

Implemented using 3DCoordX, an add-on toolbox in MorphoGraphX 2.0, meshes of 3D segmented cells can be automatically labeled and assigned to different tissue layers based on surface cells. Beside tissue annotation, 3DCoordX creates organ-based coordinates using a Bezier ring for 3D meshes, allowing analysis of highly curved multi-layer organs. This minimalizes manual work during cell annotation and allows quantitative analysis of cellular features based on organ position.

The principle behind this annotation is given in Figure 1. The initial outer L1 layer of cells (i.e. the red layer) is distinguished based upon the relative proportion of surface area that is exposed, or not adjoining another cell, termed the “outside wall area ratio”. Layers below this are then sequentially labeled based on the number of cells between them and the outer layer. Using exposed area also enables the segmentation of adjoining structures, such as neighboring ovules, even if the surface mesh of the structures is touching. The authors then place a reference point (i.e. the black dashed line) based on important developmental regulators such as gene or hormone expression.

**Figure 1:**
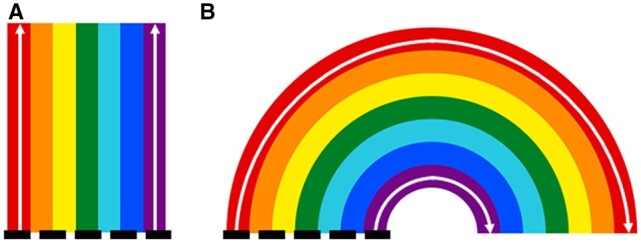
Schematic overview of tissue and cell layer annotation accounting for the growth axis as implemented by Vijayan et al. (2022) A, straight tissue structure and B, curved tissue structure. In straight tissues, the distance from a reference point (dashed line) remains constant for each cell layer (i.e. the left versus right layer). However, in a curved structure, distance will differ depending on the layer as denoted by the arrows.

Separation of the model into sequential layers with respect to the different growth axes allows for automatic analysis of cell division and expansion to understand the processes shaping morphology. This indicates different processes governing curvature across the diverse plant species studied, from preferential cell proliferation in the anterior domain of ovule primordia in Arabidopsis, to increased cell length during axial neck growth in the liverwort archegonium, and to distinct cellular patterns resulting in the folding of the complex floating bladderwort trap.

With continued advances in hardware and software, it is feasible that intelligent model annotation, as achieved here, will be replicated across other 3D models. For example, segmentation of organs at the whole plant level poses a considerable task for studying whole-plant morphological traits (Lomte and Janwale, 2017).

## Funding

A.J.B. is supported by The Leverhulme Trust.

*Conflict of interest statement*. The authors declare no conflict of interest.
